# Lightweight LiDAR-Based 3D Human Pose Estimation via 2D Depth Images for Autonomous Driving

**DOI:** 10.3390/s26051631

**Published:** 2026-03-05

**Authors:** Gyu-Yeon Kim, Somi Park, Sunkyung Lee, Bobin Seo, Seon-Han Choi, Sung-Min Park

**Affiliations:** 1 Graduate Program in Smart Factory, Ewha Womans University, Seoul 03760, Republic of Korea; happ9907@ewhain.net; 2 Division of Electronic & Semiconductor Engineering, Ewha Womans University, Seoul 03760, Republic of Korea; 1118som@ewhain.net (S.P.); snklee20@ewha.ac.kr (S.L.); qhqls28@ewha.ac.kr (B.S.); smpark@ewha.ac.kr (S.-M.P.)

**Keywords:** autonomous driving, LiDAR, 2D depth image, 3D human pose estimation, lightweight

## Abstract

Real-world traffic is highly dynamic, with pedestrians exhibiting unpredictable movements. Pedestrians’ poses are essential cues for predicting their actions, enabling vehicles to respond proactively and reduce accident risks. In autonomous driving, the distance between vehicles and pedestrians is critical, making 3D human pose estimation crucial. In this context, pedestrian pose estimation has been actively studied, and recently, light detection and ranging (LiDAR) sensors have attracted attention due to their accurate 3D depth information and privacy benefits. However, existing LiDAR-based 3D pose estimation methods mainly process 3D data directly, requiring high computational cost and memory. In this paper, we propose a lightweight LiDAR-based 3D human pose estimation method specifically designed for deployment in autonomous driving systems. Unlike conventional 3D direct processing methods, our approach strategically reduces computational complexity by projecting point clouds into 2D depth images and leveraging a lightweight MoveNet, followed by efficient 3D lifting. Furthermore, we introduce a self-occlusion correction algorithm to improve robustness under side-view and bending poses, where depth-based projections often suffer from distortion. Experimental results on benchmark datasets demonstrate that the proposed method achieves competitive pose estimation accuracy while substantially improving efficiency, highlighting its practicality and scalability for real-time autonomous vehicle applications.

## 1. Introduction

Recent advances in autonomous driving have significantly enhanced vehicle capabilities. Nevertheless, the complexity and uncertainty of real-world traffic environments continue to pose critical safety challenges. Among road users, pedestrians are particularly dynamic and unpredictable, exhibiting irregular trajectories and velocities, with behaviors that can change abruptly depending on context and individual decisions [1,2]. Therefore, beyond mere detection, understanding and prediction of pedestrian behavior are essential for safe and reliable autonomous driving. In this context, pedestrian pose provides important clues about both their actions and intentions. By observing posture and movement, it is possible to predict whether a pedestrian is about to cross the road or is approaching a vehicle, allowing vehicles to respond proactively and reduce the risk of accidents. Research has demonstrated that even predicting 30 cm in pedestrian trajectories can have a significant difference on collision risk [3], and that advancing emergency braking by as little as 0.16 s can greatly reduce injury severity [4]. In autonomous driving systems, the distance and height between vehicles and pedestrians directly influence safety-critical decisions, making 3D human pose estimation essential.

Against this background, research on pedestrian pose estimation has been predominantly driven by camera-based approaches. In particular, 2D pose and motion representations extracted from RGB images have been widely used to infer crossing intention and future motion dynamics. These representations are often integrated with temporal modeling frameworks, including graph-based spatio-temporal methods, to capture structured human motion patterns in driving scenarios [5,6,7]. Additionally, robust camera-based detection research has also been actively pursued to address challenging environmental conditions. For instance, IHENet introduces an illumination-invariant hierarchical feature enhancement network to improve object detector performance under low-light conditions [8]. Similarly, robust perception frameworks leveraging data augmentation and domain adaptation have been proposed to enhance detection reliability across adverse weather and visibility shifts [9]. Other recent detection models focus on maintaining performance under fog, rain, and nighttime scenarios.

Nevertheless, camera-based approaches inherently suffer from performance degradation under extreme illumination conditions. Moreover, camera setups lack direct and accurate distance-aware 3D geometric information, which is crucial for safety-critical decision making in autonomous driving. In addition, the capture of identifiable visual information may raise privacy concerns in public environments. These limitations have drawn increasing attention to LiDAR-based approaches, which provide accurate geometric depth measurements, operate independently of ambient lighting, and preserve structural cues without recording detailed personal appearance features [10,11].

Existing LiDAR-based human pose estimation research has primarily evolved around deep learning models that directly process point clouds in 3D space. Notably, LiDARCap [12], proposed by Li et al., established a fundamental pipeline that extracts frame-wise point features using PointNet++, incorporates temporal information through gated recurrent unit (GRU), performs joint rotation estimation based on spatio-temporal graph convolutional networks (ST-GCNs), and generates 3D human mesh via the skinned multi-person linear (SMPL) model. Following this seminal work, various subsequent studies have been proposed, including MOVIN [13], LiDARCapV2 [14], NE-LiDARCap [15], and LiveHPS [16]. MOVIN enabled more diverse pose generation by modeling pose variation distributions and combining them through a mixture of experts (MOE) architecture. LiDARCapV2 introduced the AgNoise-Segment module to improve pose recognition performance even when human point clouds are occluded by objects. Furthermore, NE-LiDARCap extended the original LiDARCap architecture to enable more robust human pose estimation by leveraging background point clouds, while LiveHPS enhanced estimation stability by introducing a knowledge distillation mechanism for scenarios involving noisy or partially occluded point clouds.

While these studies have demonstrated the feasibility of LiDAR-based 3D human pose estimation, most rely on direct 3D point processing based on PointNet++, which requires high computational cost and memory usage. This stems from multi-stage sampling and iterative neighborhood point operations [17], presenting limitations for deployment in autonomous driving environments where resource-restrained and real-time processing is essential. To mitigate these challenges, various alternative approaches have been proposed. LPFormer [18] combined 3D point features with 2D bird’s-eye view (BEV) information through a transformer architecture. VoxelKP [19] converted 3D LiDAR point clouds into voxel representations and leveraged sparse convolution-based networks. Meanwhile, LidPose [20] transformed point clouds into range-view representations and modified the ViTPose architecture to enable effective pose estimation even with sparse patterns of non-repetitive circular scanning (NRCS) LiDAR sensors.

2D representation-based approaches such as LidPose have been proposed as promising alternatives in terms of computational efficiency as a lightweight solution for 3D data processing. These methods significantly reduce computational cost by converting point clouds into range images or depth images, while enabling the utilization of mature 2D convolutional neural network architectures. However, existing 2D representation-based LiDAR research has primarily focused on object detection or semantic segmentation, showing limitations in adequately modeling the skeletal structure of human [21,22,23,24]. In the case of LidPose, the focus lies on pose estimation in sparse NRCS LiDAR sensor environments rather than lightweight implementation, and it exhibits limited performance in estimating 3D joint coordinates when LiDAR observations are insufficient or when depth information is missing due to partial body occlusion.

Consequently, while existing research has demonstrated achievements in terms of accuracy, it remains unsuitable for autonomous driving environments in terms of low-latency performance and computational efficiency. Moreover, despite the existence of lightweight approaches utilizing 2D representations, studies effectively applying these to human pose estimation have not been sufficiently presented. In particular, research on compensating for self-occlusion situations in 2D representation-based approaches remains limited. In projection-based pipelines, depth images preserve only the frontmost surface information, often compressing or distorting spatial relationships between overlapping joints. This structural characteristic makes self-occlusion more pronounced, especially in long-range autonomous driving scenarios where point density is limited. Therefore, lightweight design and robustness to self-occlusion must be addressed simultaneously for practical deployment.

Therefore, this paper proposes a lightweight LiDAR-based 3D human pose estimation method for autonomous driving environments, explicitly designed to address both computational constraints and self-occlusion issues arising from 2D projection-based representations. The main contributions are as follows:

- We present a lightweight design for LiDAR-based 3D human pose estimation that integrates 2D pose estimation with depth-based 3D lifting to achieve efficient 3D joint reconstruction.
- We propose an algorithm to compensate for self-occlusion issues that arise during projection into 2D representations. This algorithm corrects 3D joint coordinates that are lost during depth image generation. It can be extended to compensate for limitations inherent to the observational characteristics of existing LiDAR sensors.

The remainder of this paper is organized as follows. Section 2 describes the proposed lightweight LiDAR-based 3D human pose estimation method and its modules. Section 3 presents experimental setup and performance analysis. Section 4 provides conclusions and future research directions.

## 2. Methodology

### 2.1. Method Design

#### 2.1.1. Overall Structure

To achieve computational efficiency while preserving essential spatial structure for autonomous driving deployment, the proposed framework replaces computationally intensive 3D point-based processing with a 2D projection-based representation. This design enables the use of mature lightweight 2D pose estimation architectures, followed by a depth-based lifting strategy for 3D reconstruction.

**Figure 1:**
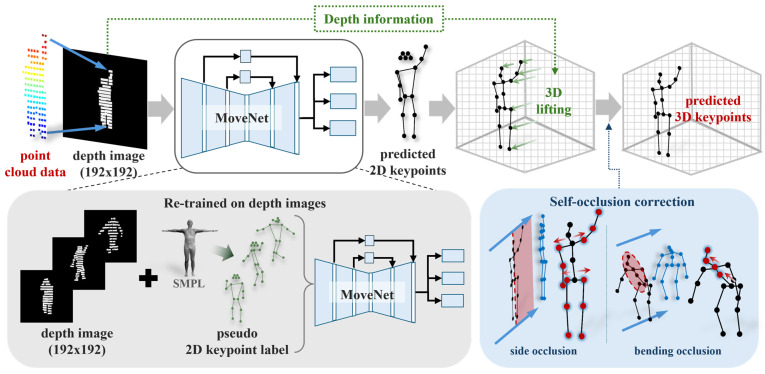
Overall structure of the proposed LiDAR-based lightweight 3D human pose estimation method. LiDAR point clouds are converted into depth images, from which 2D human keypoints are estimated using a lightweight pose estimator. The keypoints are lifted to 3D joint coordinates, followed by a rule-based self-occlusion correction module.

Figure 1 illustrates the overall structure of the proposed method. The input is human point cloud data [25], and the output is 17 3D joint coordinates. First, the LiDAR point cloud is converted into a 2D depth image to simplify the input representation. The depth image is fed into MoveNet [26], a 2D human pose estimation network, to estimate 2D keypoints for the 17 joints. These keypoints are subsequently lifted into 3D space using a depth-based lifting process, forming a simplified yet effective pipeline for 3D joint estimation.

Following the lifting stage, self-occlusion effects introduced during 2D projection are addressed through a rule-based geometric correction algorithm combined with data-driven calibration. This module compensates for distorted or missing joint positions caused by overlapping body parts or depth compression. By exploiting anatomical symmetry and statistical joint length priors, the correction process refines structurally inconsistent joints while preserving pose coherence under challenging self-occlusion scenarios.

#### 2.1.2. Depth-Based 2D Pose Estimation

The human point cloud data acquired from LiDAR is projected onto a 2D plane in the camera coordinate system, and a depth image is generated by assigning depth values to each pixel. Since point cloud data is 3D unstructured data without a grid structure, computational complexity increases during feature extraction. In contrast, conversion to a depth image allows efficient representation of 3D spatial information by maintaining the advantages of a 2D grid structure while incorporating depth information into each pixel. Moreover, research on lightweight network architecture has been actively conducted in the field of 2D image-based pose estimation, and depth images have the advantage of directly leveraging these existing 2D pose estimation models. In this study, point clouds were converted into depth images using the projection function provided by Open3D [27]. A virtual pinhole camera model was configured, and the point cloud was rendered from a fixed frontal viewpoint. The depth buffer was then captured to generate a pixel-wise depth image. The generated depth image was normalized and converted to an 8-bit image, and then cropped around the central region to match the pose estimation network input size of 192 × 192.

**Figure 2:**
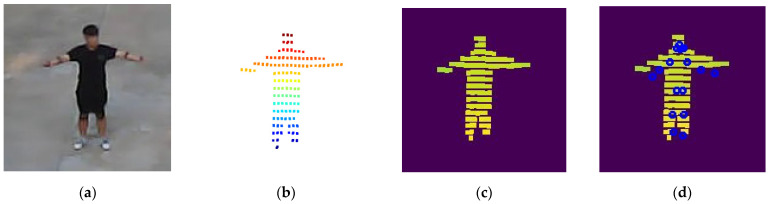
Depth image projection for 2D human pose estimation. (**a**) RGB image, (**b**) the corresponding human point cloud with color-coded depth values, where red indicates closer regions and blue indicates farther regions, (**c**) the projected depth image used as the actual input to MoveNet, where yellow corresponds to greater distances and green corresponds to shorter distances, and (**d**) 2D human keypoints estimated from the depth image, where the same color encoding is applied, with yellow indicating farther regions and green indicating closer regions.

The depth image is subsequently used as input for 2D keypoint estimation. In this study, MoveNet is employed by retraining depth images using pseudo 2D keypoint labels generated from the SMPL model. MoveNet is a lightweight pose estimation model based on convolutional neural networks (CNNs). Owing to its CNN architecture, the model effectively captures edge features of human depth. This property allows MoveNet to remain robust in depth images, where visual cues are more limited than in RGB images. Additionally, MoveNet is well-suited for real-time pose estimation and resource-constrained environments. Figure 2 illustrates the data conversion process in the proposed method.

#### 2.1.3. Depth-Based 3D Lifting

**Figure 3:**
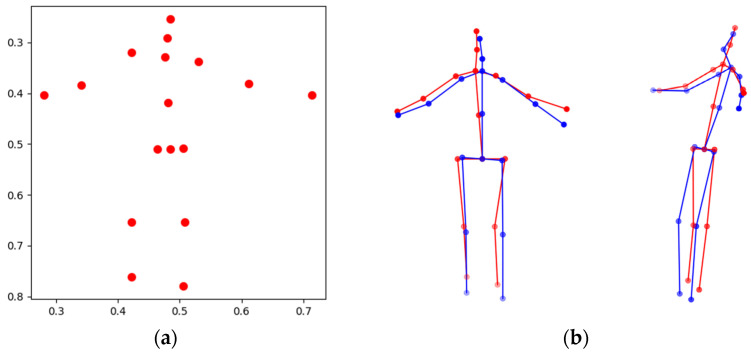
Depth-based 3D lifting of estimated 2D keypoints. (**a**) 2D keypoints estimated by MoveNet, and (**b**) corresponding 3D joint coordinates reconstructed by 3D lifting process. The ground truth is shown in blue, and the predicted pose is shown in red.

Following 2D keypoint estimation, a 3D lifting process is performed to reconstruct 3D joint coordinates by leveraging the depth information in the depth image. For each 2D keypoint, the depth value at the corresponding pixel location is assigned as the z-coordinate to form the 3D joint. Since the depth value of a single pixel may be unstable due to sensor noise or self-occlusion, depth values are sampled from a local window centered at the joint position to obtain a more reliable estimate. If valid depth information is not available, the joint depth is temporarily set to zero and later corrected using adjacent joint information to mitigate abrupt depth variations. This strategy enhances depth reliability without introducing an additional 3D regression network, enabling stable 3D reconstruction while preserving the lightweight design of the overall method. Figure 3 illustrates an example of the 3D lifting results.

### 2.2. Self-Occlusion Correction Algorithm

In a depth image, only the frontmost surface depth of the subject is recorded. This leads to a self-occlusion problem where depth information of certain keypoints is lost due to overlapping between body parts in side view poses or bending poses. In such cases, simple depth-based 3D lifting is insufficient for accurate 3D pose estimation.

**Figure 4:**
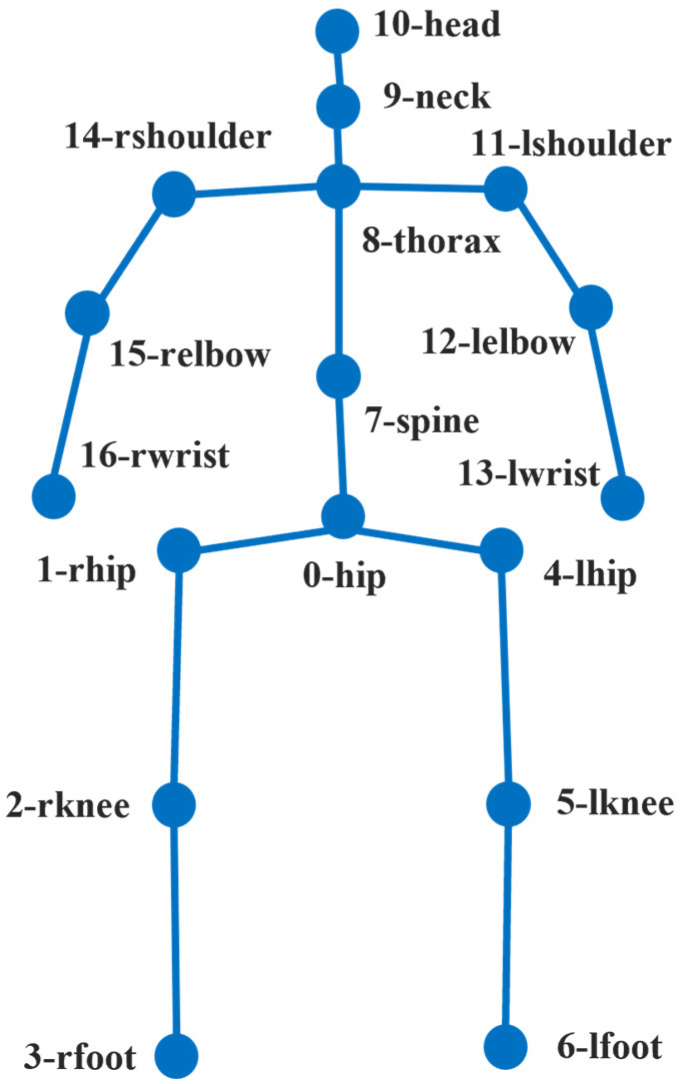
Skeleton-based design of the self-occlusion correction algorithm. H36M skeleton representation.

To address this issue, this study proposes self-occlusion correction that leverages the geometric structure of the human body based on the Human3.6M (H36M) [28] skeleton definition. Figure 4 illustrates a detailed index of the H36M skeleton used in the proposed method. Algorithm 1 illustrates corresponding pseudocode of the proposed self-occlusion correction algorithm. Algorithm 1 utilizes two structural assumptions. First, the left and right upper body joints are symmetrically positioned with respect to the spine in the H36M skeleton definition. Second, while the distances between upper body joints do not have identical absolute values, they can be modeled as reference lengths belonging to a proportional scale system. By exploiting these assumptions, the algorithm corrects the positions of joints that are occluded or distorted during depth image conversion. The algorithm is designed as a rule-based approach, enabling robust 3D joint reconstruction in self-occlusion scenarios while maintaining a lightweight method. Furthermore, due to the characteristics of LiDAR sensors, the proposed method is expected to be applicable not only to the method presented in this paper but also to other LiDAR-based 3D pose estimation models facing similar challenges.
**Algorithm 1: Self-occlusion correction algorithm**1**Input**: 3D joint coordinates $\mathrm{kp}\;\in \;R^{17\times 3}$2**Output**: Corrected 3D joint coordinates kp3spine_length $\leftarrow ||{kp}_{8}-{kp}_{7}||∕4$;4leg_length $\leftarrow ||{kp}_{0}-{kp}_{1}||∕2$5is_side ← false;6**Side-occlusion correction:**7$\mathrm{if}\;||{kp}_{11}^{xy}-{kp}_{14}^{xy}||\;<$ spine_length then8is_side ← true;9$\mathrm{if}\;{kp}_{10}^{x}\;\geq \;{kp}_{8}^{x}$ then10sign ← +1;11else12sign ← −1;13$\mathrm{if}\;||\;{kp}_{0}-\;{kp}_{1}||\;<$ spine_length then14${kp}_{1:3}^{z}\;-=\;sign\;\cdot \;spine\_length\cdot \;2.6$;15$\mathrm{if}\;||\;{kp}_{0}-\;{kp}_{4}||\;<$ spine_length then16${kp}_{4:6}^{z}\;+=\;sign\;\cdot \;spine\_length\cdot \;2.6$;17$\mathrm{if}\;||\;{kp}_{1}-\;{kp}_{4}||\;<$ spine_length then18${kp}_{1:3}^{z}\;-=\;sign\;\cdot \;spine\_length\cdot \;2.6$;19${kp}_{4:6}^{z}\;+=\;sign\;\cdot \;spine\_length\;\cdot \;2.6;$20$\mathrm{if}\;||\;{kp}_{8}-\;{kp}_{14}||\;<$ spine_length then21${kp}_{14:16}^{z}\;-=\;sign\;\cdot spine\_length\;\cdot \;2.6;$22$\mathrm{if}\;||\;{kp}_{8}-\;{kp}_{11}||\;<$ spine_length then23${kp}_{11:13}^{z}\;+=\;sign\;\cdot spine\_length\;\cdot \;2.6;$24$\mathrm{if}\;||\;{kp}_{11}-\;{kp}_{14}||\;<$ spine_length then25${kp}_{14:16}^{z}\;-=\;sign\;\cdot \;spine\_length\cdot \;2.6$;26${kp}_{14:16}^{z}\;-=\;sign\;\cdot \;spine\_length\;\cdot \;2.6$;27**Bowing posture correction:**28if is_side = false $\mathrm{and}\;{kp}_{9}^{y}\;\geq \;{kp}_{8}^{y}$ then29${kp}_{7:16}^{z}\;-=leg\_length\;\cdot \;3.4;$30${kp}_{8:16}^{z}\;-=leg\_length\;\cdot \;3.2;$31${kp}_{9:10}^{z}\;-=leg\_length\;\cdot \;1.6$;32${kp}_{10\;}^{z}-=leg\_length\cdot 1.6$;33return kp

#### 2.2.1. Side Occlusion

First, in side view poses, the body joints overlap in the viewing direction and only the depth values of the frontal body surface are recorded. As a result, the depth values of opposite body joints are compressed, and the depth structure of the body is lost. To identify side occlusion, the following condition is defined using the distance between 2D projected coordinates of two shoulders in comparison to the spine length. Spine length is defined as one quarter of the distance between the spine and thorax joints.

$$∥P_{sh}^{L}-P_{sh}^{R}{∥}_{2}<\frac{1}{4}\;∥K_{spine}-K_{thorax}{∥}_{2},$$

$P_{sh}^{L}$ and $P_{sh}^{R}$ represent the 2D projected coordinates of the left and right shoulder joints, respectively. $K_{spine}$ and $K_{thorax}$ denote the coordinates of the spine and thorax joints. When the above condition is satisfied, the pose is classified as side occlusion. This means the projected distance between the left and right shoulder joints is sufficiently reduced compared to the spine length. It indicates a high likelihood that body parts have overlapped during depth image conversion.

When side occlusion is identified, the anterior–posterior direction of the body is determined using a specific characteristic. In the H36M skeleton structure, the head joint is typically located in front of the thorax joint in natural standing poses. Therefore, the direction from the thorax to the head is used to determine the forward orientation of the body. Subsequently, the spine is set as the reference axis. The depth coordinates (z-axis) of the upper and lower body joints are then adjusted in the anterior–posterior direction. This adjustment assumes that the left and right body widths are symmetric.

The reference scale for depth correction is set as the spine length, defined as the quarter distance between the spine and thorax joints. Statistical analysis of joint lengths in the dataset revealed that a single shoulder width has an average relative length of approximately 2.6 times the spine length. Accordingly, for shoulder and hip joints, depth correction was applied using approximately 2.6 times the spine length as the reference. Furthermore, to maintain consistency in the relative positional relationships between joints, the same depth correction value is propagated to the lower joints connected to the shoulders and hips.

#### 2.2.2. Bending Occlusion

In bending poses where the upper body leans forward, the upper body joints overlap in the viewing direction and only the depth values of the frontal body surface are recorded. As a result, the depth values of the upper body joints are compressed, and the depth structure of the upper body is lost. To identify bending occlusion, the following condition is defined using the vertical positional relationship between the thorax and neck joints. This applies only to cases not classified as side occlusion.

$$\;Bending=\left\{\begin{array}{c} 1,\;\;if\;(\neg side\;occlusion)\;\cap \;(K_{thorax}^{\left(y\right)}\leq K_{neck}^{\left(y\right)})\\ 0,\;\;otherwise \end{array}\right.$$

$K_{thorax}^{\left(y\right)}$ and $K_{neck}^{\left(y\right)}$ represent the height of the thorax and neck joints in the coordinate system, respectively. This condition reflects the characteristic of bending poses where the head descends. When a bending occlusion is identified, depth correction is performed for each upper body segment. The length between right hip and hip is set as the reference scale based on joint length statistics in the dataset. Analysis results show that the hip-to-spine segment has an average relative length of approximately 1.7 times the reference length, and the spine-to-thorax segment has an average relative length of approximately 1.6 times the reference length. In contrast, the neck segment (thorax to head) above the spine has an average relative length of approximately 0.8 times the reference length. Accordingly, for the upper body where depth distortion is significant in bending poses, depth correction of approximately 1.7 times and 1.6 times the reference length is applied to the spine and thorax joints. For the neck segment, depth correction of approximately 0.8 times reference length is applied. This segment-wise correction aims to restore the upper body depth structure compressed by bending according to data-driven anatomical proportions. The corrected depth values are designed to be sequentially propagated to their subordinate joints to maintain relative positional relationships.

## 3. Experiments

### 3.1. Experimental Settings

To validate the proposed LiDAR-based lightweight 3D human pose estimation framework under autonomous driving constraints, comprehensive experiments were conducted with an emphasis on both pose accuracy and computational efficiency. Unlike prior methods that primarily pursue state-of-the-art accuracy through computationally intensive 3D point processing, our evaluation was designed to examine the trade-off between structural pose consistency and lightweight implementation. First, the 3D human pose estimation performance and efficiency characteristics were evaluated through comparison with existing models. Second, an ablation study was performed to analyze the effectiveness of the proposed self-occlusion correction algorithm.

The experiments utilized LiDARHuman26M [7], a LiDAR-based 3D human pose benchmark dataset. The dataset contains motion sequences from 13 subjects performing 20 types of daily motions in outdoor environments, with subject-to-sensor distances ranging from 12 m to 28 m. Data were captured in two scenes using fixed LiDAR sensors mounted at different heights (5 m and 7 m), resulting in diverse viewpoints and pitch angles. LiDAR point clouds were synchronized with IMU-based motion capture data, from which ground-truth SMPL parameters were obtained for single individuals. The dataset was officially split into training, validation, and test sets; in this study, we followed the official split protocol. All performance evaluations were conducted exclusively on the test set, while the training and validation sets were used only for hyperparameter tuning of MoveNet and parameter adjustment of the correction algorithm.

The accuracy was evaluated using mean per joint position error (MPJPE), Procrustes-aligned MPJPE (PA-MPJPE), and percentage of correct joints with distance to ground truth lower than 50% of the torsal length (PCK0.5).

- MPJPE: The average Euclidean distance between predicted joint coordinates and ground truth.
- PA-MPJPE: MPJPE that removed the effects of rotation and scale differences through rigid alignment.
- PCK0.5: The proportion of joints satisfying an error within 50% of the torsal length.

The lightweight characteristics and efficiency of the model were measured through the number of model parameters and frames per second (FPS). The number of model parameters was calculated using the model.parameters() function implemented in PyTorch (version 2.0.1) [29]. FPS measurement was performed in an NVIDIA RTX 3090 environment, limited to 3D pose estimation only, excluding preprocessing. While FPS values may vary depending on the hardware performance used, all methods were evaluated in the same environment to focus the analysis on relative efficiency.

### 3.2. Performance and Efficiency Evaluation

Table 1 shows the performance and efficiency of the proposed method. The experimental results show that the PA-MPJPE was approximately 108.5 mm. This confirms that relatively large positional errors exist at the individual joint level. However, the model achieved an accuracy of 89.6% on the PCK0.5 metric. This indicates that the majority of joints were positioned within an error range proportional to the body scale. These results suggest that while the proposed model has limitations in high precision joint position reconstruction, the overall skeletal structure is maintained without significant collapse. It is worth noting that our lifting process relies on LiDAR pixel depth, which measures visible surface points rather than exact joint centers. This difference can introduce systematic offsets, which may increase MPJPE and PA-MPJPE values.

To further analyze structural consistency, we report the body part-wise PA-MPJPE in Table 2. The torso region (hip, spine, thorax, shoulders, and hips) shows an error of 93.05 mm, and the head region (head and neck) exhibits an error of 88.55 mm, which are lower than the overall average. In contrast, the extremities present larger errors, with 158.28 mm for the arms and 120.05 mm for the legs. This trend indicates that the proposed model more reliably reconstructs the central body structure, while peripheral joints are relatively less precise. Since the torso and head define the global orientation and structural stability of the human body, the lower errors in these regions contribute significantly to preserving overall pose consistency. These findings indicate that the proposed model preserves the global skeletal configuration

To further evaluate the effect of sensing distance on pose estimation accuracy, we analyzed the test set by grouping samples according to subject-to-sensor distance, as summarized in Table 3. The test samples were divided into near (12–17 m), mid- (17–22 m), and far (22–28 m) ranges, and the average MPJPE and PA-MPJPE were computed for each group. The MPJPE values were 138.9 mm, 134.7 mm, and 149.9 mm for the near, mid-, and far ranges, respectively, while the corresponding PA-MPJPE values were 109.5 mm, 106.4 mm, and 115.7 mm. Notably, the mid-range shows slightly lower errors than the near range, which may be attributed to more stable point density at intermediate distances. Although slightly higher errors were observed at farther distances, the overall variation remained limited. The MPJPE difference between the mid- and far ranges was approximately 15 mm, indicating a moderate effect of distance on joint localization accuracy. This increase can be attributed to reduced point density and less detailed geometric information at greater distances. Nevertheless, the relatively small performance degradation suggests that the proposed model maintains stable structural reconstruction across varying sensing distances. These results further support that the proposed method preserves the global skeletal configuration even under changes in LiDAR observation distance.

Meanwhile, computational efficiency and structural lightweight characteristics are traded off with joints position accuracy. The total number of model parameters is 1.9 M, representing an approximately 18-fold reduction compared to the existing LiDARCap (34.93 M). The inference speed in a GPU environment also reaches 440 FPS, achieving more than a 3-fold improvement over the existing method (138.86 FPS). While existing LiDARCap-based models also demonstrate high processing speeds of approximately 138 FPS in GPU environments, their practical real-time execution is limited in CPU environments. This is due to their heavy reliance on GPU parallel computation, stemming from the point sampling and aggregation operation structure based on PointNet++.

In contrast, the proposed method is designed to enable CPU-based inference. The 2D pose estimation stage, which accounts for the largest computational portion of the entire pipeline, is structured based on the lightweight MoveNet architecture. In an actual Intel i9-12900K CPU environment, a processing speed of approximately 11.6 FPS was confirmed on a single thread basis. This CPU-based inference result demonstrates that the proposed model can operate even in environments that do not assume high-performance GPUs. This represents an important difference from existing LiDAR-based methods with high GPU dependency. Consequently, the proposed method can be utilized as a pedestrian pose estimation module for autonomous driving embedded systems or low-spec computational environments where high-performance GPU usage is difficult.

### 3.3. Ablation Work

**Figure 5:**
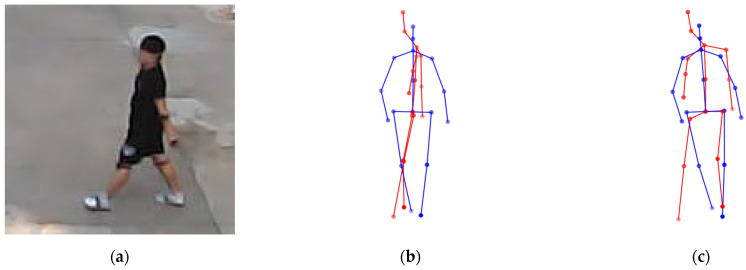
Effect of side occlusion correction in the ablation study. (**a**) The actual human pose, (**b**) pose estimation without side occlusion correction, and (**c**) pose estimation with side occlusion correction, where the ground truth is shown in blue and the predicted pose is shown in red.

**Figure 6:**
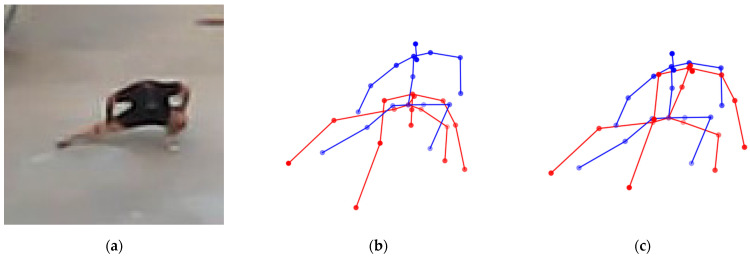
Effect of bending occlusion correction in the ablation study. (**a**) The actual human pose, (**b**) pose estimation without bending occlusion correction, and (**c**) pose estimation with bending occlusion correction, where the ground truth is shown in blue and the predicted pose is shown in red.

To analyze the effectiveness of the self-occlusion correction algorithm, an ablation study was performed by removing or adding each correction module. The base configuration represents the baseline model without any self-occlusion correction applied. Subsequently, configurations with each correction algorithm added individually or applied simultaneously were compared. Table 4 presents the MPJPE and PA-MPJPE results for each configuration. Figure 5 and Figure 6 illustrate the effects of side occlusion correction and bending occlusion correction, respectively.

When only side-occlusion correction was applied, MPJPE decreased from 152 mm to 140 mm. PA-MPJPE also improved significantly from 122 mm to 109 mm. This demonstrates that side occlusion significantly affects overall joint error. The proposed correction algorithm effectively mitigates side occlusion. In contrast, when only bending occlusion correction was applied, changes in MPJPE and PA-MPJPE were limited. However, in the full configuration where both side correction and bending correction were applied, the best performance was recorded with MPJPE of 138 mm and PA-MPJPE of 108 mm. They enable more stable pose estimation across various self-occlusion scenarios. These ablation results demonstrate that the proposed self-occlusion correction algorithm is a core component that effectively reduces structural errors in LiDAR-based 3D human pose estimation.

### 3.4. Failure Cases

Although the proposed method shows stable performance overall, we observed representative failure cases caused by front–back ambiguity in depth-only inputs.

**Figure 7:**
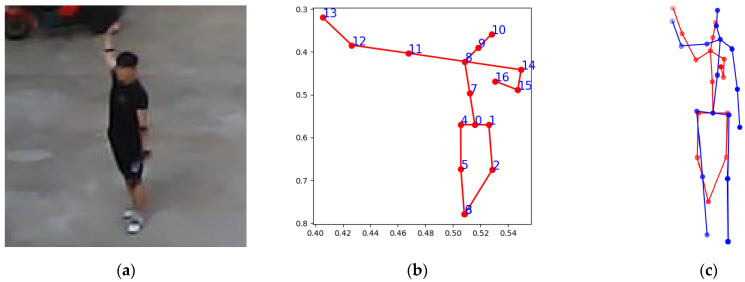
Failure case caused by front–back ambiguity in 2D pose estimation. (**a**) The actual human pose. (**b**) MoveNet fails to distinguish front and back views, resulting in flipped 2D joints; the numbers correspond to the joint indices defined in Figure 4. (**c**) The flipped 2D joints propagate to an inverted 3D skeleton after lifting. The red skeleton indicates the pose predicted by the proposed method, while the blue skeleton represents the ground-truth 3D pose.

Unlike RGB images, the converted depth images contain only human silhouettes and depth information, without texture, color, or facial cues. Under such conditions, MoveNet may incorrectly infer body orientation. Figure 7 presents a representative example of this failure. In this case, the subject is facing the sensor in the ground truth image; however, MoveNet predicts flipped 2D keypoints corresponding to a back-facing pose. Because the 3D lifting network directly relies on the predicted 2D keypoints, this orientation error propagates to the reconstructed 3D pose. Although the overall 3D pose configuration appears visually similar to the ground truth, the global orientation is inverted. Consequently, the PA-MPJPE increases significantly to approximately 221 mm in this example. This large error is primarily due to incorrect facing direction estimation rather than substantial joint localization errors. Across the entire test set, this type of orientation failure was observed in 67 out of 24,008 samples, corresponding to approximately 0.28% of the total cases.

Such front–back ambiguity is particularly critical for downstream tasks that depend on accurate orientation cues, including walking direction estimation and pedestrian intent prediction. These results suggest that depth-only, low-resolution inputs inherently limit reliable front-back discrimination, and that additional orientation-aware mechanisms or temporal consistency constraints may be necessary to mitigate this issue.

## 4. Conclusions

This study proposes a lightweight method for LiDAR-based human pose estimation by projecting 3D point clouds into 2D depth images followed by a depth-based 3D lifting process. In addition, a self-occlusion joint correction algorithm is designed to mitigate depth distortion caused by self-occlusion. Experimental results show a PA-MPJPE of 108.5 mm and a PCK0.5 accuracy of 89.6%, indicating that despite joint-level errors, the overall skeletal structure remains largely intact. The proposed model achieves strong computational efficiency with only 1.9 M parameters and an inference speed of 440 FPS on a GPU. Furthermore, applying the self-occlusion correction algorithm reduces the MPJPE and PA-MPJPE by 9.2% and 11.5%, respectively, compared to the base configuration, confirming its effectiveness.

In future work, we will investigate the robustness and generalization capability of the proposed method under sparse and low-resolution LiDAR conditions commonly encountered in real-world autonomous driving scenarios. We also aim to address body direction ambiguity in depth-only pose estimation by exploring orientation-aware modeling strategies. In addition, the self-occlusion correction module will be extended using statistical characteristics derived from more diverse populations and pose variations. Finally, the proposed method will be expanded toward scene-level multi-person tracking and interaction analysis in complex urban environments, integrating pedestrian behavior and intention prediction modules while maintaining a lightweight architecture suitable for real-time deployment.

## Figures and Tables

**Table 1:** Performance and efficiency results.

Model	MPJPE (mm)	PA-MPJPE (mm)	PCK0.5	Parameters	FPS (GPU)
LiDARCap [12]	79.31	66.72	95.00	34.93 M	138.86
LiDARCapV2 [14]	73.21	63.42	96.02	-	-
NE-LiDARCap [15]	72.23	61.67	95.79	-	-
LPFormer [18]	95.72	79.03	94.87	-	-
Ours	138.1	108.5	89.6	1.9 M	440

**Table 2:** Body-part-wise PA-MPJPE (mm).

Body Part	Joints	PA-MPJPE (mm)
Torso	hip, spine, thorax, rshoulder, lshoulder, rhip, lhip	93.05
Head	head, neck	88.55
Arms	relbow, rwrist, lelbow, lwrist	158.28
Legs	rknee, lknee, rfoot, lfoot	120.05

**Table 3:** Distance-wise evaluation results.

Distance Range (m)	Samples	MPJPE (mm)	PA-MPJPE (mm)
Near (12–17 m)	8963	138.9	109.5
Mid (17–22 m)	11,872	134.7	106.4
Far (22–28 m)	3173	149.9	115.7

**Table 4:** MPJPE and PA-MPJPE results for different self-occlusion correction configurations. The base configuration represents the model without correction modules, and “+” indicates that the corresponding correction module is added to the baseline model.

Setting	Side	Bending	MPJPE (mm)	PA-MPJPE (mm)
Base	X	X	152	122
+Side	O	X	140	109
+Bending	X	O	151	121
+Side + Bending (Full)	O	O	138	108

## Data Availability

The dataset used in this study is the publicly available LiDARHuman26M dataset. The dataset is openly accessible at https://github.com/jingyi-zhang/LiDARCap (accessed on 2 March 2026).
